# Modified Medtronic Microvascular Plug Implantation to Reduce Pulmonary Blood Flow in Patients With a Shunted Single Ventricle

**DOI:** 10.1016/j.jscai.2023.101070

**Published:** 2023-07-14

**Authors:** Lauren N. Carlozzi, Troy A. Johnston, Thomas K. Jones, Agustin E. Rubio, Brian H. Morray

**Affiliations:** Division of Pediatric Cardiology, Department of Pediatrics, University of Washington/Seattle Children’s Hospital, Seattle, Washington

**Keywords:** congenital heart disease, Medtronic Microvascular Plug, single ventricle, systemic to pulmonary artery shunt

Balancing systemic and pulmonary blood flow (PBF) in patients with a shunted single ventricle can be challenging. In patients with symptomatic excessive PBF, medical strategies are sometimes inadequate. Surgical clips or bands can be applied to reduce blood flow but require an additional operation and cannot be applied to patent ductus arteriosus (PDA) stents because of the risk of stent damage.

Transcatheter techniques are limited. The Diabolo stent technique modifies a covered stent, creating an hour-glass shape to reduce blood flow; however, it is typically performed in larger patients given the larger delivery sheath size.^1^ Several case series and animal studies have described modification of the Medtronic Microvascular Plug (MVP) (Medtronic) by removing sections of its polytetrafluoroethylene (PTFE) covering to effectively reduce the cross-sectional area of a blood vessel.^2^ To date, this technique has been largely used in branch pulmonary arteries (PA) to reduce excessive PBF or as part of a percutaneous stage 1.^3^, ^4^, ^5^

This case series describes the first use of a modified MVP to reduce PBF in patients with a single ventricle with evidence of overcirculation after palliation with either a surgical systemic-to-PA shunt or PDA stent.

## Methods

**Figure 1:**
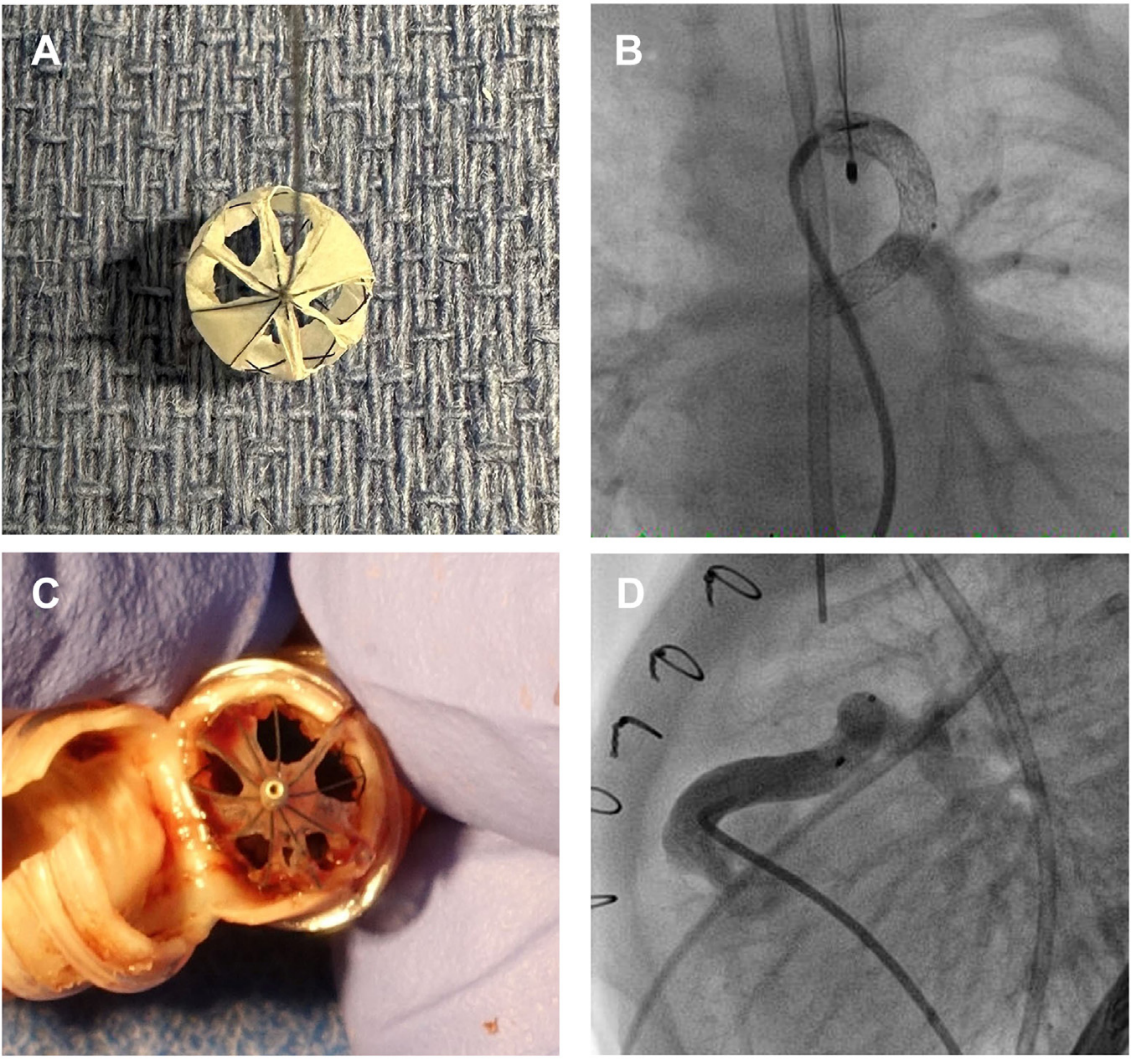
**Examples of Microvascular Plug modification in patients 1 and 3.** (**A**) A modified Microvascular Plug (MVP)-5Q with exposure of 6 of 8 covered cells for use in the 3.5-mm ductal stent in patient 1. (**B**) Angiography performed prior to release of the modified MVP-5Q within the ductal stent demonstrates flow through the device into the branch pulmonary arteries (PA). (**C**) In patient 3, after orthotopic heart transplant, pathologic examination of the 6-mm right ventricular PA conduit demonstrates the modified MVP-7Q with exposure of 7 of 10 covered cells. (**D**) Angiography performed prior to release of the modified MVP-7Q within the RV-PA conduit in patient 3.

The MVP has a self-expanding nitinol frame in 4 sizes (3Q, 5Q, 7Q, and 9Q) with an occlusive PTFE covering. Each device size is rated for a range of vessel diameters and can be deployed through a 2.7F microcatheter or 4F diagnostic catheter. Prior to deployment, incisions were created in the PTFE spanning the individual cells using a #11 blade scalpel, and the PTFE was retracted using forceps (Figure 1). The MVP-5Q has 8 covered cells, and the MVP-7Q has 10 covered cells. Between 50% and 75% of cells were exposed initially to achieve an estimated 50% reduction in cross-sectional area. Hemodynamics were monitored for 20 minutes after device deployment to determine the effectiveness of flow restriction with an expected decrease in arterial saturations by 5% to 10% in all patients and an increase in diastolic blood pressure (DBP) by 10 mm Hg in patients with aortopulmonary shunts. If this response was not achieved, the device was removed and further modified or replaced with a new device with additional cells removed. Informed consent was obtained from each family after discussing the off-label use of this device.

## Results

Three patients with symptomatic excessive PBF after single ventricle palliation were identified at Seattle Children’s Hospital between December 2019 and 2020.

### Patient 1

Patient 1 was born with pulmonary atresia and ventricular septal defect (VSD) and underwent ductal stenting with 3 overlapping 3.5-mm stents on day of life (DOL) 4. There was symptomatic pulmonary overcirculation with high saturations (85%-95%) and low DBP (20-30 mm Hg), ventricular dysfunction, renal insufficiency, and mechanical ventilatory support. On DOL 45, the patient underwent placement of a modified MVP-5Q after exposing 6 of 8 covered cells with a reduction in saturations (75%-85%) and 10 to 20 mm Hg improvement in DBP (Figure 1). Anticoagulation was initiated with enoxaparin (anti-Xa goal of 0.7-1.0), with no evidence of thrombosis. After the procedure, the patient was diagnosed with severe multilevel airway obstruction, and the family elected to pursue comfort care on DOL 63.

### Patient 2

Patient 2 was born with transposition of the great arteries, VSD, and a hypoplastic right ventricle (RV). An arterial switch operation and VSD closure was performed on DOL 3, which was complicated by extracorporeal membrane oxygenation support due to hypoxemia. The postoperative course was further complicated by PA thrombosis requiring surgical thrombectomy, complete heart block, and need for dialysis. Due to persistent hypoxia with a hypoplastic RV, a 3.5-mm Blalock–Taussig–Thomas shunt was placed on postprocedural day 8, and the patient was successfully decannulated from extracorporeal membrane oxygenation. The patient developed signs of pulmonary overcirculation with high saturations (90%-97%), mechanical ventilatory support, and diastolic hypotension requiring inotropic support (DPB, 20-35 mm Hg). On DOL 70, a modified MVP-5Q was placed in the Blalock–Taussig–Thomas shunt after exposing 4 of 8 covered cells, leading to an overall decrease in saturations (85%-90%) and 10 to 20 mm Hg improvement in DBP. The patient was previously anticoagulated with a continuous heparin infusion (unfractionated heparin goal 0.3-0.6), which was continued with no evidence of shunt thrombosis. Despite the physiologic improvement, the patient had multiple comorbidities, and the family elected to pursue comfort care on DOL 81.

### Patient 3

Patient 3 was born with an unbalanced atrioventricular canal defect and hypoplastic aortic arch and underwent the Norwood procedure with a nonvalved 6-mm RV-PA conduit on DOL 4. The patient developed severe atrioventricular valve regurgitation, was unable to wean from positive pressure ventilation or milrinone, and did not tolerate enteral feeds. The patient was listed for heart transplant. To reduce PBF while awaiting transplant, a modified MVP-7Q was placed on DOL 27. Five cells were exposed, resulting in excessive desaturation, and 2 additional cells were exposed, resulting in an appropriate reduction in saturations (75%-80%). Enoxaparin (anti-Xa goal of 0.5-1.0) was initiated for anticoagulation with no evidence of thrombosis. The patient tolerated respiratory support wean to high-flow nasal cannula at 4 L/min and feeding advancement to fortified full oral feeds and underwent successful heart transplant at 3 months of age.

## Discussion

Balancing systemic and PBF in single ventricle neonates can be difficult. This series describes the off-label use of a modified MVP as a transcatheter shunt flow restrictor in 3 neonates with symptomatic overcirculation. The MVP is a promising transcatheter option because it is easily modifiable, delivered through low-profile catheters, and recapturable, unlike the Diabolo stent, which cannot be retrieved after deployment and requires a larger, 6F catheter delivery system.^1^ Other alternatives, such as clips or bands, require an additional sternotomy and cannot be used in PDA stents due to stent distortion, making the modified MVP a more attractive option in these high-risk patients.

The extent of device modification necessary to adequately reduce PBF remains unknown. We initially modified the PTFE covering to reduce the cross-sectional area of the vessel by approximately 50%, estimating that there would be minimal flow around the device. If necessary, the device was further modified to achieve the desired physiologic outcome. Other reports have described a greater reduction in cross-sectional area, but those implants were performed in bilateral branch PA and not in patients with shunt-dependent PBF.^2^, ^3^, ^4^, ^5^ The risk of shunt thrombosis remains unknown. Each patient was therapeutically anticoagulated with higher target levels than those of typical prophylaxis given the theoretically high risk of thrombosis, especially with patients 1 and 3 having single source PBF. There was no clinical or imaging evidence of shunt thrombosis during follow-up.

There were no procedural adverse events, and the desired physiologic response was achieved in each patient. Two patients died after the procedure secondary to accumulated noncardiac comorbidities, while the third demonstrated clinical improvement and underwent successful heart transplant. This case series demonstrates that the MVP can be modified to function as a vascular flow restrictor and can reduce PBF in shunt-dependent patients. Further work is necessary to study techniques for device modification and postprocedural anticoagulation.
